# Impact of Intrinsic and Extrinsic Motivation on Work Engagement: A Cross-Sectional Study of Nurses Working in Long-Term Care Facilities

**DOI:** 10.3390/ijerph19031284

**Published:** 2022-01-24

**Authors:** Derong Zeng, Nozomu Takada, Yukari Hara, Shoko Sugiyama, Yoshimi Ito, Yoko Nihei, Kyoko Asakura

**Affiliations:** 1 Graduate School of Medicine, Tohoku University, 2-1 Seiryo-Machi, Aoba-ku, Sendai 980-8575, Japan; zeng.derong.p11@kyoto-u.jp (D.Z.); takada@med.tohoku.ac.jp (N.T.); y.hara@med.tohoku.ac.jp (Y.H.); s.sugiyama@med.tohoku.ac.jp (S.S.); y_itoh@med.tohoku.ac.jp (Y.I.); yonihei@med.tohoku.ac.jp (Y.N.); 2 School of Nursing, Kyoto Koka Women’s University, 38 Nishikyogoku Kadono-cho, Ukyo-ku, Kyoto 615-0882, Japan; 3 School of Nursing, Miyagi University, 1-1 Gakuen, Taiwa-cho, Kurokawa-gun 981-3298, Japan; 4 Department of Nursing, Faculty of Health Sciences, Tohoku Fukushi University, 1-8-1 Kunimi, Aoba-ku, Sendai 981-8522, Japan

**Keywords:** work engagement, work motivation, intrinsic motivation, extrinsic motivation, nurse, long-term care facilities

## Abstract

Nurses’ work motivation impacts their job satisfaction and work engagement, affecting their quality of care. Work motivation, a personal resource, can be categorized into intrinsic and extrinsic motivation, each of which may function differently in the job demands–resources (JD–R) model. To study the effect of nurses’ intrinsic and extrinsic work motivation on work engagement in long-term care (LTC) facilities, we randomly selected 1200 facilities from 6055 LTC facilities in eastern Japan. Two nurses from each facility completed a self-report questionnaire—newly developed for this study for evaluating intrinsic and extrinsic work motivation—to assess their work engagement, job satisfaction, and work motivation. Multiple regression analysis of 561 valid questionnaires investigated the relationship between work motivation and work engagement, indicating that intrinsic work motivation, job satisfaction, and age had a significant positive effect on work engagement, while extrinsic work motivation had no significant effect. However, half the nurses chose to work because of extrinsic work motivation, explaining the high turnover rate of nursing staff in LTC facilities. Findings indicate the importance of measures to foster nurses’ intrinsic motivation to improve work engagement. Further research should investigate how to improve the intrinsic motivation of nurses working in LTC facilities.

## 1. Introduction

Along with increasing life expectancy and decreasing fertility rates, the aging population worldwide has grown, especially in conjunction with the extensive social and economic changes taking place globally [1]. The demand for long-term care (LTC) services will increase dramatically and is likely to reach crisis levels in many countries [2]. Nurses who provide high-quality and safe medical care in LTC facilities are critically important to residents of the facilities, their family members, governments, and society as a whole [3]. Therefore, this study investigated and elucidated the relationship between nurses’ work motivation and work engagement, factors that influence the quality and safety of medical care in Japanese LTC facilities.

In Japan, a super-aged country, the LTC needs of the older population are expected to be substantial. According to Japan’s National Institute of Population and Social Security Research, the number of older adults aged 65 and above is 36.19 million as of September 2020, which is a historical high number, accounting for 28.8% of the total population; moreover, it is expected to further increase to 38.4% by 2040 [4]. The Long-Term Care Insurance System of Japan was established in April 2000 to respond to such changes in the population structure. At the time of its establishment, the number of people certified as needing LTC or support by the Long-Term Care Insurance System of Japan was 2.181 million; by the end of June 2019, the number had tripled to 6.649 million [5]. The increase in the number of older adults, the declining birth rate, and the increasing number of nuclear families make it difficult for the family to care for older adults alone. Consequently, the demand for nursing care facilities will increase in the future.

Moreover, the Japanese Ministry of Health, Labor and Welfare proposed the establishment of a Community-based Integrated Care System by 2025, which aims to comprehensively ensure that older adults can continue to live in a familiar area until the end of their lives, support independent living, and maintain dignity [6]. This calls for a shift toward increased community-based home care, as opposed to hospital-centered medical care [7]. As the number of and needs associated with LTC facilities are increasing [8], the role of nurses is becoming more critical. However, their low willingness to continue working in LTC facilities is a significant problem.

Being the only resident care staff with advanced medical training in LTC facilities, nurses play an essential role in health management, decision-making, and cooperation with regional medical institutions and families. However, the number of nurses working in LTC facilities is only 6.5% of the total number [9]. The turnover rates of nurses in general and of first-year nurses in LTC facilities specifically are 16.4% and 38.3%, respectively [10,11]. Furthermore, 43.1% of the nurses reported their intention to quit their jobs and leave the profession altogether [12]. These findings imply that Japanese nurses did not feel fulfilled by their work in LTC facilities.

The concept of fulfillment is associated with work engagement. Work engagement is defined as “a positive, fulfilling, and affective-motivational state of work-related well-being, characterized by vigor, dedication, and absorption” [13]. Vigor refers to a “high level of energy and psychological resilience during work,” dedication involves “strong involvement in work, meaning and pride of work,” and absorption refers to “concentration and immersion in work” [14].

The extant research on work engagement and work outcomes has been conducted mainly in corporate academic research. In particular, the positive relationship between employees’ work engagement and organizational outcomes has been clarified in previous studies regarding productivity [15], job performance [16,17], and economic benefits [15,18]. In addition, the job demands–resources (JD–R) theory discusses “work engagement in addition to burnout and considered burnout and work engagement to be mediators of the relation between job demands and negative outcomes (health problems), and job resources and turnover intention, respectively” [19]. Therefore, work engagement can regulate the relationship between job resources and outcomes. Job demands refer to the physical, psychological, social, or organizational aspects of work that continually demand physical and psychological (cognitive and emotional) efforts or skills. Job resources refer to the physical, psychological, social, and organizational aspects of a job that promote the achievement of goals; job resources can reduce the physical and psychological costs resulting from work demands and promote personal growth and development [20,21].

Previous research has suggested that personal resources, which are similar to job resources, play a role in the JD–R model. For example, (1) personal resources directly impact well-being [18,22]; (2) personal resources moderate the relation between job characteristics and well-being [23,24]; (3) personal resources mediate the relation between job characteristics and well-being [25,26]; and (4) personal resources influence the perception of job characteristics [26,27]. Personal resources are defined as “the psychological characteristics or aspects of the self that are generally associated with resiliency and that refer to the ability to control and impact one’s environment successfully.” [19] Especially, intrinsic work motivation, which is included in personal resources, increases job autonomy’s positive effect on work engagement [24].

The literature on nurses’ work engagement has been growing. High levels of work engagement are associated with high job satisfaction [28,29], low burnout and turnover intention [29], and low job change intention [30]. Lower work engagement results in increased turnover intention in nurses [30,31]. High work engagement impacts the organization as follows: (1) preventing nurse shortages, (2) effective use of limited medical expenses, (3) providing high-quality care [32], (4) lowering the mortality rate, and (5) improving organizational profitability [33]. Low levels of work engagement have led to a reduction in the quality of care and patient satisfaction [34]. Low work engagement also has healthcare implications, such as increased medical spending and turnover rates. Conversely, high work engagement boosts the levels of safe and high-quality medical care provided by nurses and nursing managers.

The present study focused on work motivation’s impact on the work engagement of nurses working in LTC facilities. According to JD–R theory, positive work motivation is one of the factors that corresponds to job resources, influencing work engagement [35]. Therefore, we assume that positive work motivation is a factor that enhances work engagement. Moreover, turnover intention has a negative effect on work engagement [36]. We also assume that positive work motivation is a factor that suppresses turnover intention. In other words, this study assumes that nurses working at LTC facilities with positive motivation may maintain high work engagement.

In this study, work motivation has been divided into intrinsic and extrinsic motivation. According to self-determination theory (SDT) [37,38], this division is based on the different reasons or goals that give rise to an action. Intrinsic motivation is defined as the performance of an activity for its inherent satisfaction rather than for some separable consequence. When intrinsically motivated, a person is moved to act for the fun or challenge entailed rather than external prompts, pressures, or rewards. Extrinsic motivation is a construct that pertains to an activity that is performed to attain some separable outcome, as opposed to engaging in an activity simply for its instrumental value.

Several studies have reported the effects of intrinsic and extrinsic motivation on work engagement. For example, monetary reward is one of the factors influencing extrinsic motivation for workers and can increase work engagement [39]. Career planning and performance evaluation, which are factors in extrinsic motivation, have a positive impact on work engagement [40]. Recent studies have shown that intrinsic motivation has a more positive effect on work engagement than extrinsic motivation [41]. A systematic review of studies on work engagement in nursing practice in 2016 revealed 77 factors that influence work engagement, such as age and job satisfaction [34]. However, no studies have focused on the relationship between work motivation and work engagement in the professional nursing field. Clarifying the relationship between work motivation and work engagement will improve nurses’ work engagement to ensure high-quality care for older adults in LTC facilities [42,43]. It will also provide empirical evidence that personal resources (intrinsic work motivation) play a role in the JD–R model, similar to job resources, function to help accomplish work goals, and stimulate personal growth and development. Therefore, it is necessary to study the effect of work motivation on work engagement in LTC facilities.

Job satisfaction affects work engagement [44]. Further, although age, sex, educational background, marital status, and number of children have not been consistently studied in previous studies [45,46,47], they have been shown to affect work engagement and have been used as control variables.

**Figure 1:**
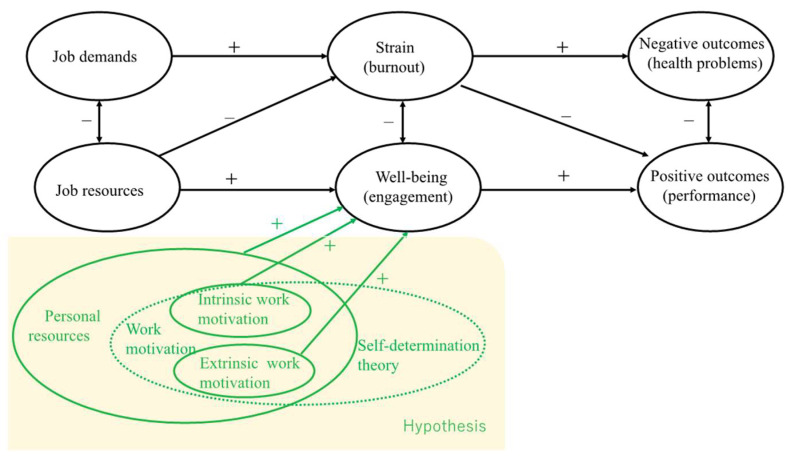
The theoretical model.

Thus, we hypothesized the following: intrinsic and extrinsic motivation to work in LTC facilities have a direct, positive impact on work engagement. Figure 1 shows the hypothetical model developed for the JD–R model and the personal resources for nurses working in LTC facilities.

## 2. Materials and Methods

### 2.1. Research Design

This study adopted a cross-sectional design. The study setting was LTC facilities (including LTC welfare facilities and LTC health facilities) located in eastern Japan and listed in the Welfare and Medical Service Network System—a nationwide online database. The sample size was calculated such that the sample is representative of the general population in terms of work engagement scores and the standard deviation of work engagement was set at 0.92. Additionally, assuming that the standard error is 1 and the reliability is 95% in this survey, the required number of participants was 328. The questionnaire was collected via Japan Post. Based on a response rate of 27% in a survey of LTC facilities in Japan [48], we assumed that the collection rate of the questionnaire would be 15–25% and estimated slightly more, thus the required number of participants was 2400. Consequently, the number of target facilities was 1200 and we decided to target two nurses (registered nurses or licensed practical nurses) at each facility, a total of 2400 nurses. Thus, 1200 facilities were randomly selected from 6055 nursing homes in eastern Japan as the sample for this research. To allow sufficient time for participants to respond to the questionnaire, we set a submission deadline of approximately two weeks after receiving the questionnaire. The questionnaires, which included information regarding the research purpose and consent to participate, were mailed to the LTC facility managers, who then distributed them to the nurses. The completed questionnaires were returned to the researchers by mail. The survey period was from February–May 2019.

### 2.2. Measures

#### 2.2.1. Demographic Variables

Sex, age, marital and child status, types of LTC facility, years of experience at the current facility, and educational background were collected as individual and work-related variables.

#### 2.2.2. Work Engagement

The Japanese version of the Utrecht Work Engagement Scale was used to measure work engagement [49]. This scale comprises nine items, each of which is concerned with vigor, dedication, and absorption. Example items are “At my work, I feel (that I am) bursting with energy” and “When I get up in the morning, I feel like going to work.” All items were rated using a seven-point Likert-type scale, ranging from 0 (“none”) to 6 (“always”). The higher the score, the greater is the work engagement.

#### 2.2.3. Job Satisfaction

Job satisfaction was measured using the Job Satisfaction Scale developed initially by Mclean [50]. It has been shown to have an effect on work engagement, as an adjustment variable in the multiple regression analysis. We used 7 of the scale’s 15 items to measure job satisfaction. Example items are “I am satisfied with my current workplace” and “I am satisfied with the content of my current job.” Responses were provided using a five-point Likert-type scale, ranging from 1 (“Dissatisfaction”) to 5 (“Satisfaction”), with higher scores indicating that the respondent was satisfied with working in LTC facilities.

#### 2.2.4. Work Motivation for Employment in LTC Facilities

**Figure 2:**
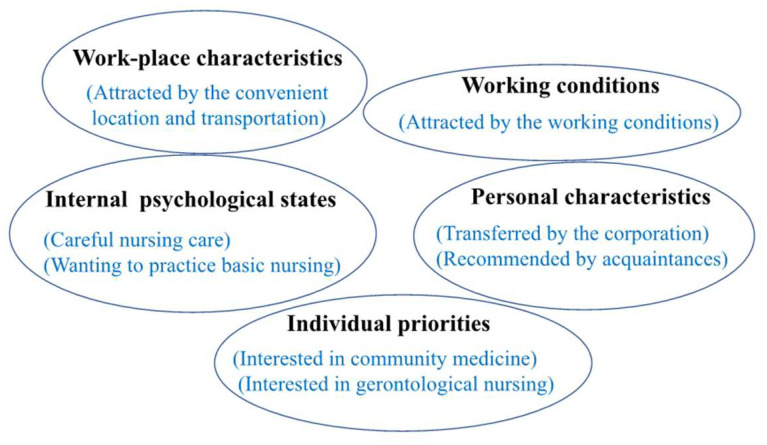
Logical validity.

From September to November 2018, three nursing management experts and four nurses with a master’s degree jointly reviewed the extant literature on work motivation. The choice of work motivation as the main variable was largely based on previous studies [51,52,53,54,55]; accordingly, we carefully selected items during the researchers’ meeting. Based on the five factors of nurses’ work motivation identified in the review literature, we developed an eight-item original questionnaire to assess nurses’ motivation to work in the current LTC facility. The items were discussed until consensus was reached. Multiple choices were allowed for all items. Figure 2 presents the five factors, which were selected based on a comprehensive literature review [55]. Therefore, it can be considered that this questionnaire is logically valid. The questionnaire was divided into four items each for intrinsic and extrinsic motivation. The questionnaire items were as follows: “I was interested in community medicine (interested in community medicine),” “I wanted to practice basic nursing (wanting to practice basic nursing),” “I was interested in gerontological nursing (interested in gerontological nursing),” “I wanted to provide careful nursing care without being overwhelmed by time (careful nursing care),” “I was transferred by the corporation, and it was not my own will (transferred by the corporation),” “I was recommended by acquaintances (recommended by acquaintances),” “I was attracted by the convenient location and better transportation facilities (attracted by the convenient location and transportation),” and “I was attracted by the better working conditions, such as no night shift and working hours (attracted by the working conditions).” Respondents were asked to select all the work motivation items from the eight-item checklist. A higher number of items selected indicated a higher intrinsic or extrinsic work motivation among nurses in LTC facilities.

### 2.3. Data Analyses

Descriptive statistics were calculated for all data, and the measures of tendency of the data were determined. The scores for work motivation were calculated, and the distribution of scores was confirmed. This study employed a multiple regression model using SPSS v23.0 J (IBM Corp., Armonk, NY, USA) for Windows to clarify the effect of work motivation on work engagement. Work engagement was the dependent variable, and the independent variables were the demographic variables, job satisfaction, and work motivation (intrinsic and extrinsic). The significance level was set at *p* < 0.05.

### 2.4. Ethical Considerations

The questionnaire and study purpose and design were distributed to the participants, who were informed that their confidentiality and anonymity would be ensured during the research and publication process. Participation was voluntary, and returning or submitting the questionnaires was deemed to indicate consent to participate. It was stated that it was difficult to withdraw consent after returning the questionnaire because of the anonymous nature of the questionnaire survey. Therefore, the deadline for submission was set with sufficient time to complete the questionnaire. This study (no. 2018-1-717) was approved by the Ethics Committee of the Graduate School of Medicine on 17 January 2018 and conformed to the provisions of the Declaration of Helsinki of 1995 (as revised in Edinburgh 2000).

## 3. Results

At the end of May 2019, 565 questionnaires were collected from the 2400 nurses. Five hundred and sixty-one completed questionnaires were included in the analysis (valid response rate: 23.4%); four were excluded because they contained missing data. Table 1 presents the participants’ characteristics.

### 3.1. Work Motivation

Table 2 shows the selection frequency of work motivation. The frequency and percentages of selecting intrinsic work motivation items were 56 (10%), 73 (13%), 230 (41%), and 113 (20.1%) for items 1, 2, 3, and 4, respectively. The frequency and percentages of selecting extrinsic work motivation items were 74 (13.2%), 115 (20.5%), 215 (38.3%), and 115 (27.6%) for items 5, 6, 7, and 8, respectively.

With regard to the number of items selected, Table 3 shows the selection ratio of intrinsic and extrinsic work motivation. For intrinsic work motivation, a total of 250 (44.6%) participants did not choose any. The proportion of participants who chose 1, 2, 3, and 4 items were 33.5%, 15.9%, 5.3%, and 0.7%, respectively. While as, for extrinsic work motivation, a majority (*N* = 294; 52.4%) chose one item, while 24.2%, 23%, 0.2% and 0.2% of the participants chose zero, two, three, and four items, respectively.

### 3.2. Reliability

In this study, the Cronbach’s α coefficient of the work engagement scale was 0.92 [49], while Job Satisfaction Scale was 0.877 [56].

### 3.3. Work Engagement

Work engagement scores are presented in Table 4.

### 3.4. Impact of Work Motivation on Work Engagement

A multiple regression analysis was performed with work engagement as the dependent variable and individual attributes (age, sex, educational background, number of children, years working at the current facility), job satisfaction, and work motivation (intrinsic and extrinsic motivation) as the independent variables. Table 5 presents the analysis results, which revealed that children (*β* = 0.095, *p* < 0.05), intrinsic motivation (*β* = 0.164, *p* < 0.001), job satisfaction (*β* = 0.375, *p* < 0.001), and age (*β* = 0.104, *p* < 0.05) were factors that had a significant positive effect on work engagement. Sex, educational background, marital status, extrinsic motivation, and years working at the current facility had no effect on work engagement.

## 4. Discussion

This study aimed to investigate the effect of the work motivation of nurses in LTC facilities on their work engagement. While intrinsic work motivation enhances work engagement, extrinsic work motivation does not affect work engagement. The originality of this study is that work motivation was divided into intrinsic and extrinsic motivation. To the best of our knowledge, this is the first study to investigate the relationship between work motivation and work engagement in professional nursing practice. Moreover, these findings have both theoretical and practical implications, as elaborated ahead.

Nurses working in LTC facilities had relatively poor work engagement levels. The mean work engagement score of nurses in LTC facilities was compared with data from previous studies to elucidate the characteristics of the study sample [57,58,59,60,61]. The mean work engagement scores in this survey were 2.98, and nurses working in LTC facilities appeared to be less engaged in their work than other occupations in Japan. Scores above 3.20 were considered high in a non-nursing study that used the same scale in the context of Japan [57]. However, many studies on nurses from other countries have reported mean engagement scores above 4 out of 6 in all three dimensions [58,59,60]. For example, a study from 185,835 Spanish nurses reported a mean engagement score of 4.59 [61]. Nevertheless, the work engagement scores tended to have positive correlations with nurses’ age in the current study, which is consistent with findings from other studies [62,63,64]. However, the relationship between age and engagement in the literature is not consistent, and more studies on this relationship are needed in the future.

Nurses working in LTC facilities had more extrinsic motivation than intrinsic motivation. In this study, work motivation included four items for extrinsic and four items for intrinsic motivation. The percentage of those who did not choose any extrinsic motivation item was 24.2%, and the proportion of those who did not choose any intrinsic motivation item was 44.6%. Furthermore, over half of the participants chose the items “Attracted by the convenient location and transportation” and “Attracted by the working conditions” under extrinsic motivation. The nurses in this study sought to achieve a balance between work and family.

The average age of the study participants was 48.8 ± 9.7; 75.2% were married, 80.7% had children, and 92% were women. In 2016, the Japan Nursing Association conducted a nationwide elderly care facility survey targeting nurses working in LTC facilities. According to this survey [65], the average age of the nurses who worked in LTC facilities was 48.15, and 92.7% were women. The attributes of the participants of this study were similar to those of Japan as a whole and to a national survey of 4945 nurses who worked in LTC facilities in Norway, having a mean age of 41.8 years, with 95% being female [66]. The findings of this and previous studies indicate that being middle-aged and married are characteristics of nurses working in LTC facilities. Their primary motivation is to balance work and family.

Additionally, since many nurses are not well acquainted with their work and since insufficient research attention has been paid to motivation in nursing homes, this may explain why intrinsic motivation is lower. Most care staff at LTC facilities in Japan and other countries are nursing assistants. For example, in Japan, 80% of the care staff at LTC facilities are nursing assistants [8], while 80.4% were nursing assistants in a multicenter cross-sectional study conducted across 105 LTC facilities in France [67]. Therefore, it is easy to recognize that care in these facilities is centered mainly on nursing assistants. For these reasons, it is not difficult to understand why intrinsic motivation for nurses working in LTC facilities is low. More research is needed in the future to increase nurses’ intrinsic motivation to work in LTC facilities.

### 4.1. Impact of Work Motivation on Work Engagement

In this study, two reasons may be considered to explain why extrinsic motivation did not significantly affect work engagement. First, 55.4% of nurses in this study chose only the following extrinsic motivation items: (1) Transferred by the corporation, (2) Recommended by acquaintances, (3) Attracted by the convenient location and transportation, and (4) Attracted by the working conditions (e.g., night shift or working hours). This means that more than half considered a balance between work and family when they chose to work in LTC facilities. Since extrinsic motivation affects job search, once this is fulfilled it no longer impacts nurses’ work engagement. On the other hand, work engagement is a concept that deals with the internal state of a worker’s psychology, and extrinsic motivation is an external factor related to behavior. Work engagement and extrinsic motivation involve different dimensions, and hence there is no significant relationship between them.

Intrinsic motivation was one of the factors that had a significant effect on work engagement, which supported the results of previous studies [34,68]. In this study, four intrinsic motivation items were investigated as follows: (1) Interested in community medicine, (2) Wanting to practice basic nursing, (3) Interested in gerontological nursing, and (4) Careful nursing care. These intrinsic motivation items are closely related to nurses’ satisfaction, joy in working in LTC facilities, and work engagement. Of the nurses, 44.6% reported having no intrinsic motivation and had lower work engagement. This indicates that it is vital to improve nurses’ intrinsic motivation to work in LTC facilities. Further research is necessary to clarify how to improve the intrinsic motivation of nurses working in LTC facilities.

### 4.2. Contributions to Nursing Practice and the JD–R Model Theory

As stated in the introduction, low levels of work engagement have reduced patient care quality, which in turn increases medical costs and turnover rates. Moreover, high levels of work engagement not only improve the quality of care but also reduce the turnover rate of nurses. The results of this study show that intrinsic work motivation has a significant impact on work engagement, and therefore nursing managers need to strengthen education, particularly on the importance of gerontological nursing and community care, associated with intrinsic work motivation.

Furthermore, this study provides empirical evidence for the role of personal resources (intrinsic work motivation) in the JD–R model, enhancing our understanding of the JD–R theory. The findings also provide a basis for the development of nurses’ work motivation theory.

### 4.3. Limitations

First, we randomly selected 1200 LTC facilities (LTC health and welfare facilities) from Japan’s 6055 LTC facilities. The findings are limited only to part of Japan and the Japanese context, which may not reflect national trends and limits the generalizability of the results. Therefore, the background and culture of the research environment must be considered when interpreting the results. Second, the method of measuring motivation for employment in LTC facilities was conducted using a self-report questionnaire. Although assessment of the scale’s psychometric properties was not part of this study, we have confirmed its validity; however, its reliability needs to be evaluated in a future study. Third, the response rates in a previous study [48] and this study were generally low. Considering deviation in the responses, measures to increase the response rate, such as developing a good cover story and instructions [69], are necessary for the future. Fourth, although concerns about methodological bias and the pervasiveness of research settings in which such bias may arise have been expressed, procedural controls are likely to be the most effective way to control common measurement biases [70,71,72]. Therefore, attention to procedural controls is recommended in future studies. Furthermore, it is also necessary to administer the same survey in other countries to compare the survey’s appropriateness in other socio-cultural contexts.

## 5. Conclusions

This study classified work motivation into intrinsic and extrinsic work motivation based on SDT theory and hypothesized that work motivation belongs to personal resources and plays a role in the JD–R model. The results showed that nurses’ intrinsic motivation to work in LTC facilities had a significant effect on their work engagement, while extrinsic work motivation had no such effect. Therefore, it can be concluded that intrinsic work motivation belongs to personal resources and has a positive effect on work engagement. However, in the context of the high turnover rate of nurses in LTC facilities, this study clarified that half of the nurses chose to work in LTC facilities based on extrinsic motivation alone. The findings of this study indicate the importance of considering measures to foster nurses’ intrinsic motivation to improve work engagement.

## Figures and Tables

**Table 1:** Participants’ basic attributes (N = 561).

Variables		N (%)
Mean age (SD+) in years		48.8 (±9.7)
Mean years working at the current facility (SD+) in years		7.7 (±6.3)
Sex	Female	516 (92)
	Male	45 (8)
Marital status	Single	100 (17.8)
	Married	422 (75.2)
	Divorced or widowed	39 (7)
Number of children	0	108 (19.3)
	1+	453 (80.7)
Types of long-term care facilities ^a^	Long-term care welfare facilities	252 (44.9)
	Long-term care health facilities	306 (54.5)
Educational background ^a^	Vocational school or junior college for registered nurses	538 (95.9)
	Baccalaureate program (four-year program in nursing) or masters’ program in nursing	21 (3.7)

^a^ Types of long-term care facility were left blank for three people. Educational background was left blank for two people.

**Table 2:** Selection of each work motivation item: Frequency and proportion of participants (N = 561).

Items		No. ^a^ (%)
Intrinsic work motivation	1. Community medicine	56 (10.0)
	2. The practice of basic nursing	73 (13.0)
	3. Gerontological nursing	230 (41.0)
	4. Careful nursing care	113 (20.1)
Extrinsic work motivation	5. Be transferred by the corporation	74 (13.2)
	6. Recommended by acquaintances	115 (20.5)
	7. Attracted by the convenient location and transportation	215 (38.3)
	8. Attracted by the working conditions	155 (27.6)

^a^ Multiple-choice item.

**Table 3:** Number of intrinsic and extrinsic work motivation items selected: Frequency and proportion of participants.

Total Number of Items Selected	No. (%) (Intrinsic Work Motivation)	No. (%) (Extrinsic Work Motivation)
0	250 (44.6)	136 (24.2)
1	188 (33.5)	294 (52.4)
2	89 (15.9)	129 (23)
3	30 (5.3)	1 (0.2)
4	4 (0.7)	1 (0.2)
Total	561 (100)	561 (100)

**Table 4:** Work engagement scores.

	N	Min	Max	Mean	Standard Deviation
Work engagement	561	0	6	2.98	0.98

The minimum value was 0, the maximum value was 6, and the average value was 2.98 ± 0.98.

**Table 5:** Impact of work motivation on work engagement.

Individual Attributes	β	P	VIF
Age	0.104	*	1.17
Sex (Male)	−0.001	0.969	1.049
Educational background	0.062	0.111	1.031
Marital status (Married)	−0.040	0.368	1.346
Number of children (1+)	0.095	*	1.400
Job satisfaction	0.375	**	1.055
Intrinsic motivation	0.164	**	1.129
Extrinsic motivation	−0.02	0.617	1.077
Years working at the current facility	0.001	0.972	1.108

Note: Dependent variable: Work engagement; Adjusted R-squared: 0.198. * *p* < 0.05, ** *p* < 0.01.

## Data Availability

The datasets used in this study are available from the corresponding author on reasonable requests.
